# What do brain-dead patients ultimately die of?

**DOI:** 10.1186/cc13655

**Published:** 2014-03-17

**Authors:** A Kawasaki, M Hotta, K Mochiduki, N Saito, M Namiki, M Takeda, T Harada, A Yaguchi

**Affiliations:** 1 Tokyo Women's Medical University, Tokyo, Japan

## Introduction

After a patient is declared brain dead, the cessation or withdrawal of therapy in Japan is quite a difficult operation because of the legal issues involved. Therapy is continued except in the case of donation of organs for transplantation from brain-dead patients until their cardiac arrest. Consequently, we may determine what the final cause of death is in brain-dead patients. Our hypothesis is that brain- dead patients ultimately die of cardiac failure.

## Methods

From January 2011 to December 2012, brain-dead adult patients in our ICU were investigated. The declaration of brain death is determined by clinical neurological examinations, electroencephalography, and the auditory brain-stem response test. Age, sex, primary diagnosis, Glasgow Coma Scale (GCS) on admission, the number of days from the diagnosis of brain death until cardiac arrest (days), the final cause of cardiac arrest, urine volume for the last 24 hours (ml), serum potassium levels (mEq/l), PaO_2 _(mmHg) and PaCO_2 _(mmHg) on the last day were evaluated. Values are expressed as mean ± SD. Data were analyzed by Kruskal-Wallis test and Mann- Whitney *U *test. *P *< 0.05 was considered statistically significant.

## Results

Of a total 1,221 patients admitted to our ICU, 37 patients (18 men, 19 women, age 65 ± 20) were clinically diagnosed with brain death. Primary diagnoses were 18 post-cardiac arrest syndromes, 14 cerebrovascular diseases, and four traumatic subarachnoid hemorrhages. GCS on admission was 4.5 ± 2.7 and days to cardiac arrest after diagnosed brain death was 8.6 ± 13.2. Urine volume for the last 24 hours was 997 ± 1,712 and serum potassium level was 4.8 ± 1.7. PaO_2 _and PaCO_2 _were 131.9 ± 80.6 and 48.9 ± 13.8, respectively. The final causes of cardiac arrest were 20 cardiac failures, 11 renal failures and six respiratory failures, although with mechanical ventilator support. The final cause of cardiac arrest due to respiratory failure had significantly longer stay in the ICU and days after diagnosis of brain death than cardiac failure or renal failure (27.2 ± 22.6 days vs. 9.2 ± 10.0 and 8.1 ± 4.6, *P *< 0.05; 23.2 ± 24.0 vs. 6.0 ± 9.0 and 5.4 ± 5.2, *P *< 0.05, respectively).

## Conclusion

There were significantly longer days until final cardiac arrest that was caused by respiratory failure than by cardiac failure or renal failure. Many different observations in each case were shown, such as urine volume or potassium level on the last day. Not only cardiac failure but also other failures might lead to final cardiac arrest.

